# Premonitory Urges and Sensorimotor Processing in Tourette Syndrome

**DOI:** 10.3233/BEN-120308

**Published:** 2013

**Authors:** Sangeerthana Rajagopal, Stefano Seri, Andrea Eugenio Cavanna

**Affiliations:** ^1^The Michael Trimble Neuropsychiatry Research GroupDepartment of NeuropsychiatryBSMHFT and University of BirminghamBirminghamUK; ^2^School of Life and Health SciencesAston Brain CentreAston UniversityBirminghamUK; ^3^Sobell Department of Motor Neuroscience and Movement DisordersInstitute of Neurology and University College LondonLondonUK

**Keywords:** Tourette syndrome, tics, premonitory urges, sensori-motor processing, pathophysiology

## Abstract

Most patients with Tourette syndrome report characteristic sensory experiences (premonitory urges) associated with the expression of tic symptoms. Despite the central role of these experiences to the clinical phenomenology of Tourette syndrome, little is known about their underlying brain processes. In the present article we present the results of a systematic literature review of the published studies addressing the pathophysiological mechanisms of premonitory urges. We identified some preliminary evidence for specific alterations in sensorimotor processing at both cortical and subcortical levels. A better insight into the brain correlates of premonitory urges could lead to the identification of new targets to treat the sensory initiators of tics in patients with Tourette syndrome.

## 1. Introduction

Tourette syndrome (TS) is a highly prevalent neuropsychiatric disorder, estimated around 1% in school-age children [1]. According to DSM-IV criteria, the onset of tics is before 18 years of age, typically 3–8 years for motor tics and 11 years for phonic tics [2, 3], lasting at least a year. Multiple motor and vocal tics vary in localisation over time and peak in severity at around 10–12 years [4–6]. Interestingly, although first described and popularised by Georges Gilles de la Tourette, an earlier publication by Armand Trousseau seems to offer a description which is more representative of our modern day TS construct with minimal sampling bias [7]. In clinical practice the distinction between tics and habits or repetitive behaviours can be challenging, as all are characterised by various levels of awareness and volitional control [8].

Premonitory urges (PUs) are not part of the DSM-IV criteria for TS despite the vast majority of patients (77% of patients over 13 years) experience these symptoms [9–12]. PUs, also called sensory tics, are described as focal or generalised intrusive feelings or sensations driving the individual to seek relief through performance of movements (motor tics) or vocalisations (vocal tics) [13–16]. A study by Leckman et al. [17] found 10 years to be the mean age of PUs awareness, averaging 3 years after tic onset. The awareness latency is suggested to reflect a transition of the processing of sensory information to conscious awareness [18,19]. Studies with the Premonitory Urge for Tics Scale (PUTS), a psychometric tool specifically developed to measure the severity of PUs [20,21], have highlighted that awareness of PUs can be a maturational process, independent of tic onset. The most frequently reported localisations of PUs are the palms, shoulders and throat, although 40% of patients localise them exclusively in the muscle, and the remainders in their joints or skin. The percentage of patients with TS or other chronic tic disorders experiencing PUs varies depending on how the urge is defined [21–23].

ISSN 0953-4180/13/$27.50 © 2013 – IOS Press and the authors. All rights reserved

It has been proposed to categorise PUs as sensory (SUs), cognitive (CUs) or autonomic urges (AUs) [24]. SUs are focal or generalised muscular-skeletal or visceral-sensations, whereas CUs are feelings of incompleteness or 'just-right' perceptions and AUs overlap with symptoms of autonomic dysfunction, such as sweating, palpitations and nausea. Interestingly, urges can also be bound to external stimuli e.g. in automutilatory tics where specific angulation of objects can trigger tics, which are characteristically suggestible by both audio and visual cues [3]. The exact role and significance of PUs is at present unknown. It has been postulated that they could reflect subjective experiences of neural dysfunction below tic-production threshold or heightened attention to physical sensations [8]. PUs may be an illumination of fragments of innate behaviour, closely involved in the orchestration of behavioural programmes. Central to this idea is the binding role of the basal ganglia to allow movement execution in sight of convergent information from the functionally distinct cortico-subcortical circuits, which can account for the heterogeneity of TS symptoms [25, 26].

Importantly, about 90% of patients with TS can also present with co-morbid behavioural problems, mainly attention-deficit and hyperactivity disorder (ADHD), obsessive-compulsive disorder (OCD), affective disorders and impulse control disorders [27–29]. With this in mind, clinical subdivisions may be appropriate, from 'pure TS' (simply motor and vocal tics) to 'full-blown TS' (tics plus coprophenomena, echopenomena and/or paliphenomena) and 'TS-plus' (with psychiatric co-morbidities) [30]. PUs have been reported to cause more distress to patients with TS than tics, which appear semi-voluntary in response to inner urges [4, 31–33]. Self-awareness of these subjective symptoms could improve the ability to suppress tics, as shown by the effectiveness of behavioural strategies for tic control such as exposure and response prevention and habit reversal therapy [17,34,35].

Due to their intrinsic subjectivity the neural correlates of PUs are difficult to investigate. However a better understanding of their pathophysiology might have significant clinical implications. In the present article we present the results of a systematic literature review of the published studies addressing the pathophysiological mechanisms of PUs.

## 2. Methods

We conducted a systematic literature review according to the Prisma guidelines [36] using the search terms 'tic*', 'tourett*', 'anxiety', 'mechanism', 'urge*', 'sensor*' across the scientific databases EMBASE, HMIC, Medline and PsycINFO. We excluded from the review studies that did not focus on human subjects and all non-English literature. Additional relevant publications were identified by snowballing reviewed papers; "grey" literature was retrieved from Google searches using the above-mentioned search terms.

## 3. Results

Our systematic literature review identified five studies specifically focussing on the pathophysiology of PUs in patients with TS and these are summarised in Table 1. Two articles were neurophysiological studies (focussing on the Bereitschaftspotential and Somatosensory Evoked Potentials respectively), two were neuroimaging studies (structural and functional magnetic resonance imaging) and one was a recent review paper.

## 4. Discussion

### *4.1. The pathophysiology of premonitory urges*

Despite their central role in subjective tic phenomenology, PUs have received little attention and few studies have focused on this intriguing phenomenon in patients with TS. The reviewed literature highlights several hypotheses on the pathophysiological bases of PUs. Converging evidence supports the presence of sensory gating dysfunction. Specifically, gating dysfunction in TS may cause excessive inflow of somatosensory information, possibly generating PUs by acting on particular cortico-striatal synapses through LTP and increasing SMA activation. Abnormal dopamine function seems also to be implicated. Dopamine transporter hyperactivity can play a central role, as dopamine can have relevant effects on particular cortico-striatal synaptic strengths. Moreover, the striosomal compartment of the striatum is interconnected with limbic structures and can modulate matrix activity via dopaminergic neurons from the substantia nigra-pars compacta (SNpc), influencing motivation-based behaviour. Of note, there is considerable overlap with the neurobiological bases of reward experiences, which involve the mesolimbic dopaminergic system and prefrontal cortical regions interlinked with the ventral striatum. Anatomically, convergence and topographical organisation of functionally related

Table 1

Studies on the pathophysiology of premonitory urges in patients with Tourette syndrome

| Study | Aim | Hypotheses generated from study | Participants | Main findings | Limitations |
|---|---|---|---|---|---|
| Duggal et al., 2002 [31] | To investigate BPs (assessed using EMG and EEG recordings) as a measure of PUs | BPs may be affected by dopaminergic dysfunction within cortico-striatal circuitries | 2 TS+OCD, 1 CTD | Tics are more similar to intentional rather than responsive movements, but still not purely voluntary as BPs onset latency was shortened in all patients. BPs can be used as a marker of PUs | – No control group<br>– Small sample size<br>– Possible confounding role of comorbid OCD (PUs more prevalent in patients with TS+OCD)<br>– Did not compare findings with BPs assessed in voluntary movement |
| Sowell et al., 2008 [63] | To assess cortical thinning in relationship with tic severity using T1-weighted MRI | Specific cortical changes may be associated with TS | 25 TS (9 TS+ ADHD/OCD), 25 age and sex matched controls | Patients with TS have significant cortical thinning in bilateral ventral frontal cortex e.g. ventral pre and postcentral gyri. Thinning of inferior left primary sensory cortex, temporal and parietal cortices not found in younger children but present in older children and adolescents. Correlations between worst tic severity (as per YGTSS) and cortical thickness were significant in the dorsal frontal cortex bilaterally, left dorsal parietal lobes, pre- and post-central gyri, right ventral frontal cortex and right temporal cortex. Grey matter thinning within sensory areas of the fronto-subcortical circuitry, with extension to frontal and parietal cortices, may be related to PUs generation | – Correlations with tic severity (YGTSS scores) rather than PUs severity |
| Bohlhalter et al., 2006 [42] | To assess patterns of brain activity before and during tic expression and imitation using event-related fMRI | SMA, ACC, posterior putamen activation prior to tics may in part be responsible for PUs generation | 10 TS, 4 TS+OCD, 2 TS+ADHD | Before tic onset, premotor and SMA showed most significant activity, along with anterior cingulated cortex (ACC), insular region, posterior putamen, parietal operculum and ventrolateral thalamus. Their activity receded on initiation of tic movement. At tic onset, most signal came from sensorimotor, cerebellum and DLPFC. The SMA, ACC insular and thalamus seem to be involved in urge generation. The parietal operculum may allow integration of visceral and somatosensory information through its connections with the ACC and motor regions | – As tics are highly suggestible, tics performed in the scanner may be different from those expressed in the everyday environment |

Table 1, continued

| Study | Aim | Hypotheses generated from study | Participants | Main findings | Limitations |
|---|---|---|---|---|---|
| Miyazaki, 2007 [62] | To investigate SEP using median nerve stimulation in children with ADHD and tic disorders | ADHD and tic disorders, both being hyperkinetic disorders, may have common somatosensory pathology | 18 ADHD, 18 tic disorders (10 TS), 10 healthy controls | 7/18 children with ADHD and 9/18 children with tic disorders had SEP abnormalities, e.g. latency or amplitude deviations. Peak-to-peak latencies were mostly reduced and peak amplitudes were greater than controls. These findings suggest hyperactive sensorimotor fronto-subcortical circuit loops, consistent with PU symptomology. The high SEP amplitudes seem to be associated with shorted cortical silent period, suggestive of motor inhibition dysfunction | – Included all tic disorders, not just TS |
| Jackson et al., 2011 [65] | To investigate comparisons between pathological urges (e.g. in TS) and everyday urges leading to physiological spontaneous movement (e.g. yawning, swallowing) | Urges to perform movement may be related to the strength of afferents that lead to awareness of urges | Systematic literature review on the urge-for-action | Converging evidence suggests that the insular cortex and cingulate cortex may be involved in PU generation. The insular cortex registers whether the urge for an action is satisfied whereas the insular cortex, basal ganglia and cingulate cortex are involved in reward experience following urge satisfaction | – fMRIs results are difficult to compare between different studies |

Abbreviations. BPs = Bereitschaftspotentials, EMG = Electromyography, EEG = Electroencephalography, PUs = Premonitory Urges, TS = Tourette Syndrome, OCD = Obsessive-Compulsive Disorder, CTD = Chronic Tic Disorder, MRI = Magnetic Resonance Imaging, ADHD=Attention-Deficit Hyperactive Disorder, YGTSS = Yale Global Tic Severity Scale, fMRI, = Functional MRI, SMA=Supplementary Motor Area, ACC = Anterior Cingulate Cortex, DLPF=Dorso-Lateral Prefrontal Cortes, SEP = Somatosensory Evoked Potentials.

cortico-striatal projections entertain particular striatal regions while impressing functional subdivision of the striatum. The dorsolateral striatum is predominantly 'somatosensory'-related , the intermediate portion 'associative' and the ventromedial 'limbic', which allows these areas to be implicated in different aspects of movement and complex behaviour [37,38].

### 4.2. Sensorimotor gating

The most accredited pathophysiological theory emerging from this review, suggests that PUs are the expression of a 'sensorimotor gating' dysfunction. Sensorimotor gating is responsible for the modulation in the allocation of neural resources in the presence of competing sensory and cognitive information from the multitude present in the environment [39]. When premotor and prefrontal cortices initiate a motor pattern, the striatum may allow initiation if agreeable with activity of other convergent corticostriatal inputs, while suppressing competing motor programmes. Therefore, basal ganglia may be involved in selection of motor and behavioural patterns within appropriate context [40] via this gating mechanism. One study [37] found that externally paced movement created increased activity in the anterior caudate-putamen, a possible pivotal site for this 'gate' mechanism. Additionally, electrical stimulation of the SMA in non-TS individuals produced an urge to move ($n = 13$) or unusual sensations ($n = 9$) [41]. This suggests SMA's involvement in movement intentions, especially as the SMA and the anterior cingulate cortex (ACC) have dense dopaminergic projections from midbrain structures such as the substantia nigra (SN) and its activation prior to tic onset can potentially generate PUs via limbic-motor connections [42]. In relation to these pathways, other results show low metabolic activity of the ventral striatum and limbic regions and increased activity in SMA and sensory association regions [5].

### 4.3. Structured event complexes

The orbitofrontal-cortex (OFC) is typically associated with social behaviour, emotion and reward, and has been proposed to interact with BG in reward learning. Via sensorimotor gating, the BG can favour initiation of particular 'cognitive pattern generators' in the cortex [43]. Through reinforcement by dopamine influencing long-term-potentiation (LTP) [44] of particular synapses, gating can activate rewarding behaviour. The amygdala, coding for stimulus intensity, also appears to be involved. The prefrontal cortex (PFC) appears to have 'structured event complexes' (SECs)/behavioural patterns that are hierarchical in nature. In OCD models, the ACC seems to play an important role for motivation and error processing in calculation of disparities between expected and gained reward [43]. Therefore, motivation to complete a SEC could be processed through communication between reward regions such as the OFC, activation threshold assigned by the BG, error calculation by ACC and emotional importance by limbic areas. However, prevention from performing SECs is punishing and this punishment is only removed once the behaviour is completed. In OCD models, little relief is experienced because of ACC signalling incomplete behaviour, OFC punishment, anxiety generated in limbic structures and the BG reducing thresholds for compensating SECs. This 'feeling of incompletion' of SECs causes an obsession to gain relief from anxiety. However, in OCD, only a small proportion of the full reward is experienced. A similar process may occur to generate PUs in TS: tics may occur because of the reduced inhibitory BG output because of its reduced threshold possibly due to fewer inhibitory-interneurons [45] and low metabolic activity [5]. The cognitive-psychophysiological model of TS [8] suggests that patients with TS have particular cognitive styles governing 'correct' manners to undertake and organise activities - a perfectionist style. This mechanism, together with physiologically heightened sensory awareness, creates tendencies to attempt too much and reduced relaxation, paralleling ADHD [46]. This in turn creates conflicts between what should be undertaken and what is being done, diminishing feelings of achievement [8].

Interestingly, it seems that PUs may not be the only manifestation of fronto-striatal dysfunction in TS. An investigation undertaken by Eddy et al. [47] into executive function and Theory of Mind in TS revealed alterations in these functions generated by the frontal cortex. Patients and controls were assessed for reticence and verbal fluency in various tasks such as evaluating mental states by looking at images of faces, understanding types of humour and other decision processes. On comparison with controls and even with exclusion of co-morbid OCD, performance in TS was reduced, suggesting dissociation between ventromedial prefrontal cortex and the striatum. The results of different studies corroborate these findings [48,49]. Moreover, an MRI study by Draganski et al. [50] found adults with TS to have anterior cingulate, ventrolateral prefrontal and orbitofrontal grey matter volume reduction. Reduced grey matter in orbitofrontal region is thought to be related to reduced impulse control and behavioural inhibition, which are known features of TS [30]. Draganski et al. also found increased dorsolateral putamen volume bilaterally, especially in the subgroup of patients with TS+ADHD, and increased thickness in the left primary somatosensory cortex, with a significant correlation to PU severity as measured by the PUTS scores. PU sensory information triggering motor tics could potentially result in increased volume evidenced in the dorsolateral putamen via plastic remodelling through dorsolateral putamen connections to sensorimotor areas allowing 'sensory-guided' movement. Additionally, neuroleptic exposure could also result in striatal volume increases, and increased thickness of somatosensory cortex can be related to increased myelin thickness [51]. This suggests that PUs may impose greater functional demands for integration into motor responses (tics), allowing plasticity-mediated volume change. Alternatively, reduced parietal operculum (a secondary somatosensory region) thickness may cause 'rerouting' of its demands to the somatosensory cortex as a compensatory mechanism.

### 4.4. Prepulse inhibition

Weak stimuli preceding startle stimuli can cause reduction in sensitivity to sensory stimuli, resulting in reduced startle reflex magnitudes . This is termed prepulse inhibition (PPI) and is thought to be linked with basal ganglia's role in sensorimotor gating processes, shaped by genetic, developmental and hormonal factors [39]. Reduced PPI in TS appears to correlate with the presence of interfering cognitive, motor and sensory information [52]. Interestingly, this is not specific to TS, but is also found in other neuropsychiatric conditions, e.g. Huntington's disease and schizophrenia [22]. On prepulse+pulse experiments, mixed D1/D2 agonist apomorphine increased the startle magnitude and reduced PPI, possibly reflecting genetic differences in D1 and D2 receptor sensitivity [39]. Converging evidence suggests that brain structures involved in PPI may incorporate parallel connections involving limbic areas, ventral striatum and pallidum, with the cortical-subcortical projections overlapping the startle circuit at the level of the reticularis pontis caudalis [53]. Lesions of the dorsal striatum (somatosensory component) have also been shown to result in PPI reduction [54]. The PPI model suggests parallels between tics and adapted startle reflexes [55], in line with the learning model of TS [47], where distressing events may create a reflex which later develops into a reinforced tic [56].

### 4.5. The dopamine transporter system

Overactivity of the dopamine transporter system appears to be consistently implicated in PUs generation [57]. A study using amphetamine challenge in 7 patients with TS found that mean putamen intrasynaptic dopamine levels were increased by 21% [58]. However, PET scans revealed no significant differences between D2-receptors density between TS and control brains. This may be explained by low tonic (basal) DA-levels, presynaptically regulated by D2 and D3-receptors (autoreceptors). Findings of low extrastriatal D2 receptors could also point to tonic/phasic dopamine dysfunction [59]. These findings are consistent with the clinical observations of tic exacerbations triggered by environmental stimuli (e.g. stress and anxiety), leading to increased phasic dopamine release and orbitofrontal and mesolimbocortical dopaminergergic dysfunction. From a developmental perspective, aberrant habits can result from imbalances between dorsolateral-somatosensory, intermediate-associative and ventromedial-limbic striatum. These abnormalities in the prefrontal-ventral-striatal circuitry can in turn cause inappropriate behavioural or motor sequence expression, subjectively experienced as PUs.

### 4.6. Striatal tonically active neurons

Striatal tonically active neurons (TANs) are thought to play a key role in the application of motivational contexts to conditioned sensory-instigated behaviour, executed on anticipation of reward [57,66]. These neurons are cholinergic interneurons which are activated by expectation of reward. Interestingly, TANs in the caudate and putamen appear to differ in their encoding of motivational outcomes: they can influence motivation for behaviour, 'go-response' for actions with expected motivational contexts, or both, thus allowing anticipation of rewarding or undesirable circumstances with initiation of 'goal-directed behaviour.' Additionally, TANs are responsive to dopaminergic SNpc inputs, which is a likely participant in reward calculations with inputs from the thalamus [60,64]. Within the context of sensorimotor gating dysfunction in TS, TANs may facilitate formation of abnormal motivational contexts for reward anticipation leading to PUs. Fast-spiking-GABA-interneurons (FSNs) can also be implicated, as they receive somatosensory cortex projections. Synchronised oscillatory TAN and FSN firing in conditioned behaviour can modify the activity of their respective striatal projections and favour dopaminergic plasticity of particular striatal synapses possibly involved in PUs generation. According to one hypothesis on the pathophysiology of TS, specific matrix interneurons may dissociate from normal oscillatory rhythm and 'besiege' individuals with sensory experiences [36,46]. Both abnormal activity of striatal interneurons [45,60] and hyperpolarisation of particular thalamocortical neurons coupled with irregular GPi firing, may interfere with normal cortical activity and result in PUs as experienced by patients with TS [61]. However, the abnormal cortico-subcortical loop activation may also be of cortical origin, consistent with shortened cortical silent period [60], abnormal sensory-evoked potentials [62] and thinning of premotor and sensorimotor cortical areas [3,38,63].

### *4.7. Future perspectives*

Further research is needed to better characterise and understand PUs in patients with TS. Little is known about specific PUs mechanisms underlying different tic subtypes, e.g. whether PUs associated with complex or simple tics or tics in particular anatomical locations have different pathophysiological substrates. It is possible that different neural pathways are identified between different tic subtypes, beyond shared sensorimotor processes. Moreover, studies comparing mechanisms of PUs generation in TS to other disorders such as OCD and schizophrenia may reveal more details about pathophysiological differences, which could also have clinical implications in terms of effectiveness of antipsychotic treatment. Investigations into asymmetry of basal ganglia structural changes in neurodevelopmental disorders such as TS could reveal indications of brain maturation dysfunction. This could in turn have implications for structures involved in PUs generation. Likewise, investigation into whether dysfunctional development of selective cognitive abilities leads to PU awareness may be important. Finally, the possible relationship between PU severity and Bereitschaftspotential also deserves further research, in order to use neurophysiological evidence to better characterise the clinical distinction between voluntary and involuntary actions in response to PUs.
